# Hearing status among Norwegian train drivers and train conductors

**DOI:** 10.1093/occmed/kqt114

**Published:** 2013-11-07

**Authors:** A. Lie, M. Skogstad, T. S. Johnsen, B. Engdahl, K. Tambs

**Affiliations:** ^1^Department of Occupational Medicine and Epidemiology, National Institute of Occupational Health, PO Box 8149 Dep, N-0033 Oslo, Norway,; ^2^NSB Occupational Health Service, Oslo, Norway,; ^3^National Institute of Public Health, Oslo, Norway.

**Keywords:** Noise-induced hearing loss, train drivers, train conductors.

## Abstract

**Background:**

There is a general perception that train drivers and conductors may be at increased risk of developing noise-induced hearing loss.

**Aims:**

To study job-related hearing loss among train drivers and train conductors.

**Methods:**

Audiograms from train drivers and train conductors were obtained from the medical records of the occupational health service of the major Norwegian railway company. The results were compared with audiograms from an internal control group of railway workers and an external reference group of people not occupationally exposed to noise. The monaural hearing threshold level at 4kHz, the mean binaural value at 3, 4 and 6kHz and the prevalence of audiometric notches (≥25 dB at 4kHz) were used for comparison.

**Results:**

Audiograms were available for 1567 drivers, 1565 conductors, 4029 railway worker controls and 15 012 people not occupationally exposed to noise. No difference in hearing level or prevalence of audiometric notches was found between study groups after adjusting for age and gender.

**Conclusions:**

Norwegian train drivers and conductors have normal hearing threshold levels comparable with those in non-exposed groups.

## Introduction 

Noise-induced hearing loss (NIHL) is one of the most common occupational health disorders and accounts for about 60% of occupational health disorders reported to the Norwegian Labour Inspection Authority [1]. However, the main cause for hearing loss is increasing age [2]. Age-related hearing loss is somewhat more evident in men than in women [3,4]. In addition, smoking, high blood pressure, diabetes, high cholesterol level, the use of ototoxic medication and exposure to ototoxic chemicals may have detrimental effects on hearing. Leisure time noise exposure, such as from music players, and the use of firearms, power tools, chain saws etc. may also affect hearing [5].

Train personnel such as drivers and conductors are occupationally exposed to noise. The exposure level is dependent on the quality and the maintenance of the train and track. Noise measurements in the railway company that we have studied reveal average 8-hour noise exposure levels of 70–80 dBA and 70–85 dBA for train drivers and conductors, respectively. Train conductors may be more exposed than drivers while moving between carriages and performing shunting of carriages, with peak exposures of 130 dBC, or blowing the whistle on the platform (115 dBC).

There is a general perception among train drivers and conductors in the rail company in question that they are at risk of developing NIHL. There is, however, conflicting evidence for this in the literature [6–9].

Under national legislation, Norwegian doctors are obliged to report occupational disease, even when suspected, to the Norwegian Labour Inspection Authority. Consequently, several cases of NIHL have been reported to the authority by the occupational health (OH) service of the major Norwegian railway company (NSB). Based on individual assessments, as many as 45% of the train conductors and 60% of train drivers had an audiogram compatible with the criteria for reporting NIHL to the authority, namely (i) hearing loss of ≥25 dBA at either 3, 4 and 6kHz, or ≥20 dbA for all of 3, 4 and 6kHz, worse ear (not adjusted for age) and (ii) a sufficiently high workplace noise exposure level [10].

The aim of this cross-sectional study was to describe the hearing status of NSB train drivers and conductors and to compare the results with an internal non-exposed control group of railway workers and with data from a Norwegian reference population (The Nord-Trøndelag health study HUNT) [4].

## Methods

Railway employees, including train drivers and conductors, have their hearing tested periodically as part of mandatory health assessments required by national and European Union regulations for railway safety personnel. In order to be certified, an audiometric test must be done initially at the pre-employment examination and subsequently periodically at intervals of 1–5 years, depending on age. All the tests are conducted by NSB’s OH service.

The participating subjects’ last audiogram from the period 1994–2011 was obtained from the electronic medical records of the OH service together with information on age, gender and type of job.

Audiograms from train drivers and train conductors were compared with audiograms from non-exposed railway office workers who served as an internal control group. Additionally, the study population was compared with a general population control group of Norwegian employees aged 20–64. The external control group in this study comprised subjects from a screened part of a Norwegian population-based study (HUNT) [4].

The audiometric tests were performed manually by trained nurses using Madsen Xeta Otometrics pure tone audiometric testing and a THD-39P earphone headset in a soundproof booth at frequencies of 0.25, 0.5, 1, 2, 3, 4, 6 and 8kHz. The audiometer was calibrated every second year according to the requirements of the equipment provider. The examinations were performed according to the standard procedures prescribed by the Labour Inspection Authority [10].

The hearing thresholds at 4kHz for each ear and the mean of the hearing thresholds at 3, 4 and 6kHz, both ears, were computed for the exposed railway workers and for both reference groups. The frequencies 3, 4 and 6kHz were chosen since these are the frequencies most sensitive to NIHL [11]. Age and gender-specific median values and 90 percentiles were computed and compared with the reference values. Mean differences were calculated and significance tested using analysis of variation.

Since audiometric notches are regarded as an indicator of NIHL [12], the prevalence of notches was also assessed. We defined a notch as a hearing loss of ≥25 dBA at 4kHz and a difference in hearing loss between 4kHz and 2 and 8kHz of ≥10dB [13]. The odds of having a notch was calculated by binary logistic regression adjusting for age and sex.

The data analysis was performed using SPSS (IBM SPSS Statistics Version 20).

## Results

Audiograms were available for 1567 train drivers, 1565 train conductors and 4029 non-exposed railway office workers. The external control group comprised 19 795 Norwegian employees (3389 women and 16 406 men). After exclusion of subjects with previous noise exposure and ear disease, audiograms were available for 3059 men and 11 953 women.

**Figure 1.**
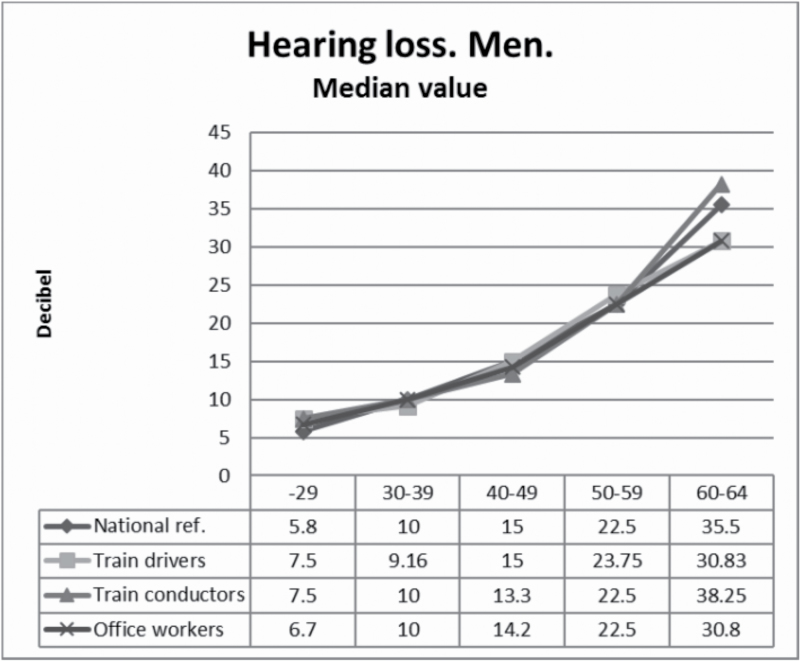
Median hearing loss at 3, 4 and 6kHz in train drivers and train conductors compared to non exposed to noise.

**Figure 2.**
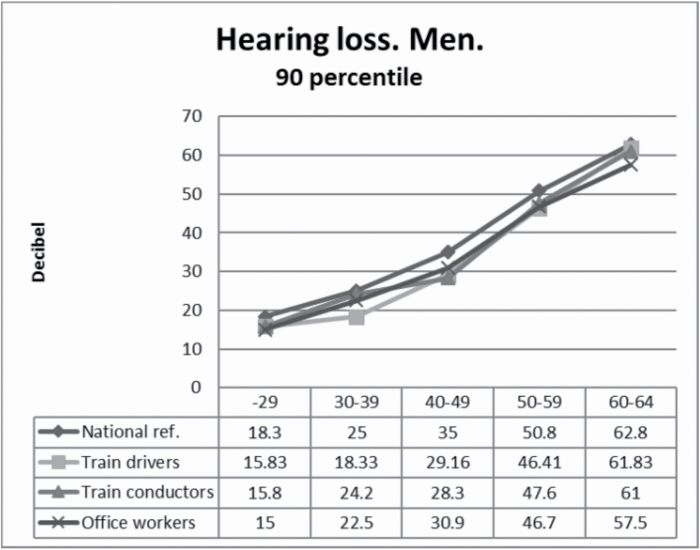
Hearing loss at 3, 4 and 6kHz in train drivers and train conductors compared to non exposed to noise. 90 percentile.

Differences in male median values, stratified by age, are shown in Figure 1. The absolute differences between any group in any stratum younger than 60 years did not exceed 1.7 dB. In the oldest group, conductors showed a higher median hearing loss (38.3 dB) than the national reference group (35.5 dB) and higher than the railway office workers (30.8 dB), but the number of conductors between 60 and 64 was very small (*n* = 58) and the difference was not statistically significant. Train drivers did as well as the office workers (30.8 dB). The 90 percentile values for men were no worse for train drivers or conductors than for the reference groups (Figure 2).

Table 1 gives an overview of the train drivers, train conductors and non-exposed office workers with respect to age, gender, noise exposure, hearing loss and prevalence of audiometric notches. Train drivers were older and included fewer females compared with conductors. The females in the three groups were significantly younger than their male colleagues.

**Table 1.** Age, gender, exposure to noise and hearing loss in train drivers (*n* = 1567) and conductors (*n* = 1565) compared with non-exposed railway workers (*n* = 4029)

	Internal reference	Train drivers	Train conductors	*P*
*n*	4029	1567	1565	–
Gender (% females)	29	8	40	<0.001^a^
Age, years: mean (SD)				
Males	45.8 (8.11)	46.1 (3.12)	40.5 (7.11)	<0.001^b^
Females	38.5 (1.11)	38.6 (3.11)	34.2 (2.10)	<0.001^b^
Occupational noise exposure (dBA)	<70	70–80+ peak	70–85+ peak	–
Hearing loss (mean 3, 4 and 6kHz binaural, dB)				
Males	20.4	20.8	17.2	<0.001^b^
Females	12.1	11.5	10.3	<0.001^b^
Adjusted hearing loss (mean 3, 4 and 6kHz binaural, dB)^c^	17.5	17.7	18.0	NS^c^
Adjusted hearing loss (4kHz, right ear, dB)^c^	16.3	16.4	16.3	NS^c^
Adjusted hearing loss (4kHz, left ear, dB)^c^	17.9	18.4	18.0	NS^c^
Prevalence audiometric notch (≥25 dB at 4kHz) (%)	9.4	12.5	7.7	<0.001^a^
Audiometric notch, OR (95% CI)^d^	1.0 (Ref)	1.09 (0.91–1.32)	1.10 (0.88–1.37)	NS^d^

OR, odds ratio; CI, confidence interval; SD, standard deviation; NS, not significant.

^a^χ^2^ test.

^b^Analysis of variation.

^c^Analysis of variation, adjusted for age and gender.

^d^Binary logistic regression adjusted for age and gender.

The unadjusted hearing loss was significantly more pronounced in train drivers and the internal control group of office workers compared with train conductors and so was the prevalence of audiometric notches. Adjusted for age and gender, the differences in hearing loss between the three groups disappeared, as did the audiometric notches (Table 1).

## Discussion

This study found that train drivers and train conductors in the study population had a pattern of hearing loss comparable with that in the two control groups not exposed to noise after adjusting for age and gender. The observed hearing loss was mainly dependent on increasing age, and males tended to be more vulnerable than females. There seemed to be no increased risk of NIHL in train drivers and conductors under normal circumstances, contrary to what we previously believed based on assessments of individual workers. However, this may not be the case in other countries where working conditions for train drivers and conductors may be different and noise exposure levels may be higher. This finding is in accordance with the understanding that average daily noise exposure levels below 85 dBA should not cause any hearing loss [14].

These results support previous negative findings concerning occupational hearing loss in railway workers [6] as well as among airline pilots and cabin attendants, who have a similar exposure to noise [15,16].

This study has a number of strengths, including the large sample sizes. We consider the audiometric measurements to be of good quality due to the training of personnel, the equipment used and the routines for calibration. Since audiometric testing is mandatory, the participation rate among both the exposed workers and controls is close to 100%. The use of control data, both from an internal control group and from other Norwegian workers, strengthens the study.

However, there are also some limitations in the study. Our assessment is based on only one audiogram (the most recent) from each participant. The cross-sectional design could introduce selection bias; for instance, certain health requirements, including normal hearing and vision and absence of various diseases, must be satisfied for train drivers and conductors to be certified fit. Therefore, some selection at recruitment might be expected. The health requirements for drivers and conductors are however very similar to those for the control group of railway workers. We therefore believe that selection factors are of minor importance.

Possible confounders have not been assessed since no information on factors other than noise that may modify hearing loss, such as smoking, high blood pressure, metabolic syndrome, diabetes and exposure to ototoxic medication, chemicals or leisure time noise were available. We have no reason to believe that these factors have had an impact on our results, since differing prevalence in the control and exposed groups studied seems unlikely.

We are lacking exposure measures, in terms of number of years of exposure, for all groups. Most train conductors and drivers are however recruited relatively young and have a low turnover rate. The same is the case for the railway office personnel. We cannot rule out the possibility that some members of these groups may have had a previous job with occupational noise exposure, but the likelihood of this influencing the results seems low since most workers join the company at an early age.

Prior to this study, there was a general perception that Norwegian train drivers and conductors are at increased risk of developing NIHL. Individual assessments of workers and their audiograms using the diagnostic guidelines of the Norwegian Labor Inspection Authority suggested that as many as 45–60% have NIHL.

It may be difficult to distinguish between noise-induced and age-related hearing loss based on audiograms only. Audiometric notches may give some guidance [14], but such notches are also prevalent in workers without any noise exposure [17]. Consequently, mandatory reporting of NILH may be of limited validity and value if the criteria for identifying such hearing loss are insufficiently robust.

In conclusion, this study has not detected any higher rate of hearing loss among Norwegian train drivers and conductors compared with non-exposed workers in the same company and with national audiometric reference values.

**Key points**

- Norwegian train drivers and conductors have audiograms comparable with controls without significant occupational noise exposure.
- Risk assessments based on reported cases of noise-induced hearing loss only are of limited validity because of the difficulty of differentiating between noise-induced and age-related hearing loss.
- The use of a non-noise-exposed reference population is of great value when assessing the risk of noise-induced hearing loss in noise-exposed working populations.

## Conflicts of interest

None declared.
